# The Book World of Medicine and Science

**Published:** 1904-07-16

**Authors:** 


### The Book World of Medicine and Science.

The Knights Hospitallers in Scotland and their Priory at Torphichen. By George Thomas Beatson, M.D., C.B. (Glasgow: James Hedderwick and Sons. 1903. Pp. vi. and 33. 8vo. Illustrated. Price 2s. 6d. net.)

Dr. Beatson tells the story of the Knights Hospitallers in Scotland and their Priory at Torphichen in a little book which is both well written and well illustrated. The Knights Hospitallers, or Knights of the Order of St. John of Jerusalem, took their origin early in the eleventh century, when some merchants of Amalfi, near Naples, erected at Jerusalem an hospice for the use of pilgrims to the holy city. The duties of the hospice were carried out at first by a band of serving brothers who devoted themselves to the work without taking monastic vows. In time they became uncloistered monks known as Hospitallers, or the white-cross knights, because they wore a black robe with a white Maltese cross. They tended the poor in houses of refuge and the sick in hospitals, gaining a great reputation for themselves by their devotion to those wounded when Jerusalem was attacked and captured in 1099. Hitherto they had been a fraternity of serving brothers, but in this year they added a military element to their duties—undertaking to protect pilgrims both in Palestine and in the journeys to and from it. The additional work was marked by a change of title, and the fraternity became the Knights Hospitallers of the Order of St. John of Jerusalem, their original black robe being covered by a red surcoat with a plain white cross whenever they were arrayed for battle. The lust of fighting grew, and to their monastic vows the Hospitallers soon added a solemn engagement to do battle with the infidel, anywhere and everywhere. The Order was grouped into three classes—the knights of noble birth, the chaplains, the serving brothers. The Hospitallers vied with the templars, and the Teutonic knights, as is known to all readers of the Crusades; but the three great orders of chivalry were driven out of Palestine, and the Hospitallers passed first to Cyprus, then to Rhodes, Sicily, and Viterbo. In 1530 the Emperor Charles V. bestowed on them the island of Malta, where they remained until 1798, when they surrendered to Napoleon. In 1827 the order was revived in England as the order of St. John of Jerusalem and it has become widely known by its affiliated branch of the St. John's Ambulance Association, founded in 1877. In their palmy days the Hospitallers were divided into Provinces or Langues, eight in number, and each presided over by a Grand Prior. England and Ireland each possessed a Grand Prior, but there was no prior for Scotland though a settlement of the order was made at Torphichen, in the west of Linlithgowshire as early as 1124. The Torphichen Priory was of considerable size and its importance is shown by the fact that it is said to have held possessions in eight of the counties of Scotland. The preceptor or head of the priory bore the title of "Lord of St. John," or "Lord St. John of Torphichen," and after the dissolution the priory possessions were made into a barony carrying with it the title of Lord Torphichen.

The Physiological Feeding of Infants. By Eric Pritchard, M.D. Second Edition. (London: Henry Kimpton. 1904. Pp. 202. Price 3s. 6d. net.)

For some years we have been acquainted with the percentage method of feeding infants, which was originated by Dr. Rotch, of America, but which has not secured much hold in this country. Dr. Pritchard is an enthusiast for this method of feeding, and gives the details of the method in full. He has recognised that the system is a somewhat complicated one and has tried to simplify it, so that the milk may be compounded at home, and the necessity for a special milk laboratory may be avoided. The intention is good, but we fear that even thus simplified there are few ordinary nurses in this country capable of carrying it out in its entirety. Chemical accuracy is the point on which great stress is laid, but biological value must also be considered in the artificial feeding of infants. It is claimed by the author that not only in health, but also in disease, the modification of milk on these lines will be the most successful line of treatment. The second part of the book is occupied by an account of the development and physiology of infancy, including a description of "the ideal infant," and "the degenerate infant." This is apparently intended to be a course of elementary instruction for mothers and nurses, and fulfils that purpose well.

Lectures, Chiefly Clinical and Practical, on Diseases of the Lungs and the Heart. By James Alexander Lindsay, M.A., M.D., F.R.C.P. (London: Baillière, Tindall and Cox. 1904. Pp. 447. Price 9s. net.)

Professor Lindsay has been well advised to issue in convenient book form the collection of lectures which during the past 15 years have been in substance delivered to his students at the Royal Victoria Hospital, Belfast. These discourses are essentially clinical: they are eminently suited to the needs of the modern practitioner. The lectures, although not systematic in point of form, are designed to raise and discuss many of the problems of diagnosis, prognosis, and treatment which daily confront the busy practitioner in the field of thoracic disease. The work is entirely practical, and the clinical standpoint is maintained throughout. The two opening lectures are of a somewhat general character, dealing with diagnostic methods and the interpretation of history in disease. The major portion of the volume is devoted to the consideration of pulmonary affections. The sections on physical examination of the lungs and pleura give abundant evidence of the independence of thought and thoroughness in action of the author. Dr. Lindsay's work throughout is written in a peculiarly attractive way, free from stultifying dogmatism and yet by no means lacking in a vigorous presentation of personal opinions. Every lecture shows evidence of wide clinical experience, painstaking research and an extensive knowledge of the best literature of the subjects dealt with. The most important and certainly the most interesting lectures are those which treat of the various aspects of pulmonary tuberculosis. Here, as elsewhere, the work is characterised by a judicious enthusiasm and a sane optimism which should go far to make it useful and popular. Professor Lindsay writes with wise discretion on the so-called hygienic treatment of phthisis. Much credit is given to Dr. Henry MacCormac, of Belfast, but no reference is made to the equally important pioneer work of such men as Bodington and the late Sir Benjamin Ward Richardson. The last eight lectures deal with cardiac affections and afford a concise and fairly complete expression of our modern views on the clinical features of cardio-vascular lesions. The practice of presenting a summary at the head of each chapter of the matters discussed in the succeeding pages is one which is likely to prove of real service. We have no hesitation in commending this work to practitioners as well as to specialists, since it is calculated to do much to stimulate and strengthen accuracy and thoroughness in the clinical investigation of chest cases.

